# Editorial: Country Profile of the Epidemiology and Clinical Management of Early Childhood Caries

**DOI:** 10.3389/fpubh.2020.00141

**Published:** 2020-04-30

**Authors:** Morenike Oluwatoyin Folayan, Maha El Tantawi, Francisco Ramos-Gomez, Wael Sabbah

**Affiliations:** ^1^Department of Child Dental Health, Obafemi Awolowo University, Ile-Ife, Nigeria; ^2^Department of Pediatric Dentistry and Dental Public Health, Faculty of Dentistry, Alexandria University, Alexandria, Egypt; ^3^School of Public Health, University of California, Los Angeles, Los Angeles, CA, United States; ^4^Faculty of Dentistry, Oral & Craniofacial Sciences, King's College London, London, United Kingdom

**Keywords:** early childhood caries, prevalence, policy, structural determinant, elimination, sustainable development goal

Early childhood caries (ECC) is the presence of decayed (cavitated and non-cavitated), filled and missing teeth due to caries, affecting the primary dentition in children less than 72 months old (1). It is the most common non-communicable disease in children (2) and a global endemic problem with those socially disadvantaged (ethnic minorities, immigrants, those of low socioeconomic status or from resource-limited settings) being most affected. The negative impact of ECC on the quality of life, growth, social development, and neurodevelopment of affected children makes it ethically imperative that public epidemiological and clinical management of ECC improves (3). Whether treated or not, ECC is a high-risk factor for caries in the first permanent molar, as highlighted by Songur et al. in this topical issue. Four other manuscripts in this special issue emphasize the urgency of addressing the endemic ECC problem. Musinguzi et al. highlighted that the prevalence of ECC in rural Uganda was 48.6% in 3–5-year-olds, and Castillo et al. showed it was as high as 76.2% in 3–5-year-olds in Peru. Also, Pierce et al. reported a prevalence of 98% in some parts of Canada, and Amalia et al. reported a prevalence of 100% in South Kalimantan, Indonesia.

Twenty years after establishing the definition of ECC, we know a lot more about ECC prevention and management (4), but we still know little about cost-effective integrated management of ECC that can control the disease using life course approaches appropriate for various cultural settings. The World Health Organization guidance document (2) promotes integration of oral healthcare into existing primary care systems. In this special issue, Villalta et al. describe how an integrated primary care model improved the knowledge and attitudes of caregivers regarding child oral health care, and Castillo et al. discuss policies, taxes, and guidelines on ECC management and labeling of food sugar content that are addressing the huge ECC problem in Peru.

Policies and taxes are strong tools for addressing structural barriers and enhancing actions for disease control, as we have learnt from tobacco control programs (5). Increased availability of ECC data will help improve the design and implementation of context-specific public oral health responses to control the disease through which we can learn about effective public health ECC control responses. The epidemiological profiles of ECC in Canada (Pierce et al.), Indonesia (Amalia et al.), Israel (Shmueli et al.), and Peru (Castillo et al.) that were presented in this issue add to our understanding of the risk factors for ECC.

Eradication—the permanent reduction of global incidence of ECC to zero—seems ambitious at the present time. However, elimination—the sustained reduction of ECC incidence to zero or levels defined by global oral health authorities—seems possible. Countries such as Finland (0.3%), Nigeria (2.7%), Kuwait (3.0%), Japan (3.9%), Tanzania (5.2%), and Sweden (6.0%) with low ECC prevalence for 0 to 2-year-old children; and Denmark with a low prevalence of 6.3% for 3 to 5-year-old children (6) may serve as global learning centers on best practices for ECC elimination.

The sparse evidence on structural determinants of ECC risk will limit efforts for ECC elimination. For ethical reasons, randomized clinical trials that might answer critical questions on structural determinants of ECC cannot be conducted; thus, the causal pathways for a multitude of inter-related factors for ECC can only be modeled (7). In this issue, Markovic et al. provide ecological data from Serbia, which suggests that structural factors like social and health care expenditures, unemployment rates, population density, and availability of physicians and dentists are determinants of the prevalence and treatment of ECC. Joury provided data suggesting that the risk for ECC in Syria increased during war and crisis, which is contrary to findings of historical studies that found a decrease in caries during war and crisis (8).

When the elimination of ECC becomes the focus of public oral health response for children under the age of 6 years (9), the World Health Organization will have to refine its targets, including setting global goals for ECC (10). Also, indicators to measure achievement should set elimination goals for 0 to 2-year-old children separate from those for 3 to 5-year-old children as emerging evidence suggests that the epidemiological profile and risk factors for ECC for the two age groups differ (6). In addition, goals and interventions for eliminating ECC should recognize that those most vulnerable to the disease are the less privileged who may be left behind if right-based interventions are not developed (11). Sadly, the populations at increased risk for ECC are likely to have no data on ECC available (6).

The 11 articles published in this topical issue provide information that can help inform policy development, policy review, and program design for the control of, and possible elimination of ECC. They contribute to our understanding of what we can modify to attain goal 3.1 of the Sustainable Development Goal for children below 6 years by 2030. The articles also provide evidence on parent-targeting interventions that can be effective (Al-Batayneh et al.; Razeghi et al.; Villalta et al.). Despite all we know about ECC, we still need more data like these published in this issue, to enable planning for control and possible elimination of ECC.

## Author Contributions

MF developed the first draft of the manuscript. ME, FR-G, and WS revised and made intellectual contributions to the subsequent drafts. All authors approve of the final version of the manuscript and for its submission.

## Conflict of Interest

The authors declare that the research was conducted in the absence of any commercial or financial relationships that could be construed as a potential conflict of interest.
